# The Stability Evaluation of Ceria Slurry Using Polymer Dispersants with Varying Contents for Chemical Mechanical Polishing Process

**DOI:** 10.3390/polym16243593

**Published:** 2024-12-22

**Authors:** Sohee Hwang, Jihee Park, Woonjung Kim

**Affiliations:** Department of Chemistry, University of Hannam, Daejeon 34430, Republic of Korea; naturalj.sohee@gmail.com (S.H.); eoutvs0725@naver.com (J.P.)

**Keywords:** chemical mechanical polishing (CMP), ceria slurry, ceria nanoparticle, polymer dispersant, stability test, removal rate (RR), uniformity

## Abstract

The chemical mechanical polishing/planarization (CMP) is essential for achieving the desired surface quality and planarity required for subsequent layers and processing steps. However, the aggregation of slurry particles caused by abrasive materials can lead to scratches, defects, increased surface roughness, degradation the quality and durability of the finished surface after milling processes during the CMP process. In this study, ceria slurry was prepared using polymer dispersant with zinc salt of ethylene acrylic acid (EAA) copolymer at different contents of 5, 6, and 7 wt% (denoted as D5, D6, and D7) to minimize particle aggregation commonly observed in CMP slurries. Among them, the D7 sample exhibited smaller particle sizes compared to commercial ceria slurry, which was attributed to the influence of the carboxyl groups (-COOH) of the polyacrylic acid polymer coating the ceria particles. It is believed that the polymer dispersant more effectively adsorbs onto the particle surfaces, increasing electrostatic repulsion between particles and thereby reducing particle size. Furthermore, the stability of the prepared slurry was evaluated under extreme conditions over three months at 25 °C (both open and closed conditions), 4 °C, and 60 °C. The D7 slurry remained stable with no significant changes observed. In addition, the prepared D7 ceria slurry exhibited a slightly higher removal rate (RR) and better uniformity, which can be attributed to the smaller particle sizes of the ceria nanoparticles compared to those in the commercial slurry. This suggests that the colloidal stability of the D7 ceria slurry is superior to that of the commercial ceria slurry.

## 1. Introduction

Chemical mechanical polishing (CMP) is a wafer surface smoothing technique that utilizes a wet process for integrated circuit (IC) chip manufacturing process. The CMP process is applied to produce many IC generations of nano-meter node, or those of even narrower line widths for a better performance [1]. This method integrates both chemical reaction and mechanical forces to achieve thorough flattening of various materials on the wafer surface. These materials include metals, dielectrics, polymers, and other thin films typically associated with manufacturing semiconductor [2,3,4,5,6].

**Figure 1:**
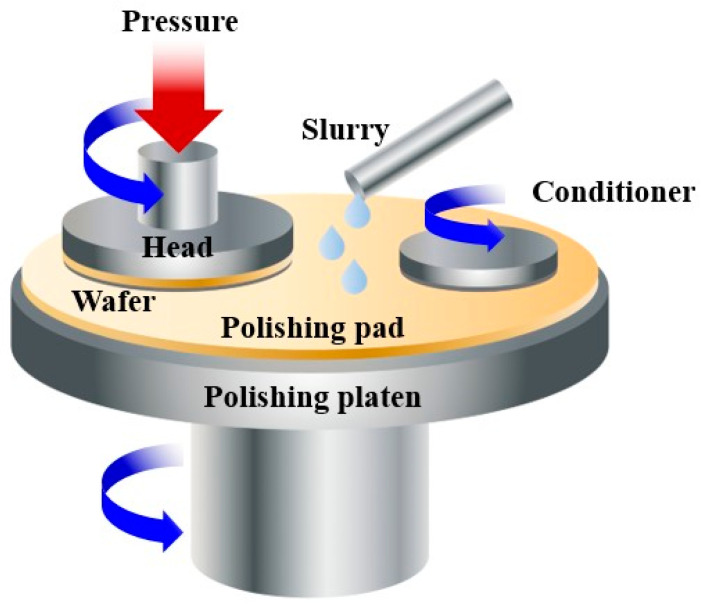
Schematic illustration of the CMP process using slurry with abrasive particles.

The ultimate goal is to create a smooth and even surface suitable for the subsequent mounting of semiconductors on the wafer. The CMP process demands a sophisticated manufacturing technology that simultaneously incorporates the chemical composition of the selected slurry and mechanical elements [7]. To obtain proper material removal rates (RR), good uniformity and low defect counts are important for slurry [8,9,10]. Therefore, selecting the appropriate abrasive and polymer dispersant within a CMP slurry is essential, as it directly impacts RR and selectivity. Conventional CMP process occurs at the interface between the surface on wafer and the polishing pad, applied pressure. the polishing pad is covered with a slurry containing particles as abrasive, and the wafer is moved across the pad to grind the surface as refer to Figure 1 [11]. The slurry is an essential and critical component. It is an abrasive particle which affects the RR, uniformity, defects, and selectivity for the materials on the wafer surface. Especially, scratches and defects on the surface of the wafer can be easily caused by the agglomeration of particles acting as abrasives. [8,9,10]. Abrasives often consist of colloidal silica (SiO_2_), fumed silica, colloidal ceria (CeO_2_), or other inorganic polishing particles that are seamlessly integrated into CMP slurry [3,12,13].

Typically, ceria particles with advantages such as low hardness and high chemical activity, have been used as the abrasive particles for the CMP process. Basim’s group found that the wafer surface damages are positively related to particle sizes and the ratio of rough abrasive particles in slurry [14]. Meanwhile, due to the capillary force in the ambient ceria abrasive particles usually stick together to form agglomerates, making it difficult to achieve direct dispersion. Therefore, it is critical to uniformity disperse CeO_2_ abrasive particles during the preparation and modification of CMP slurry [15].

**Figure 2:**
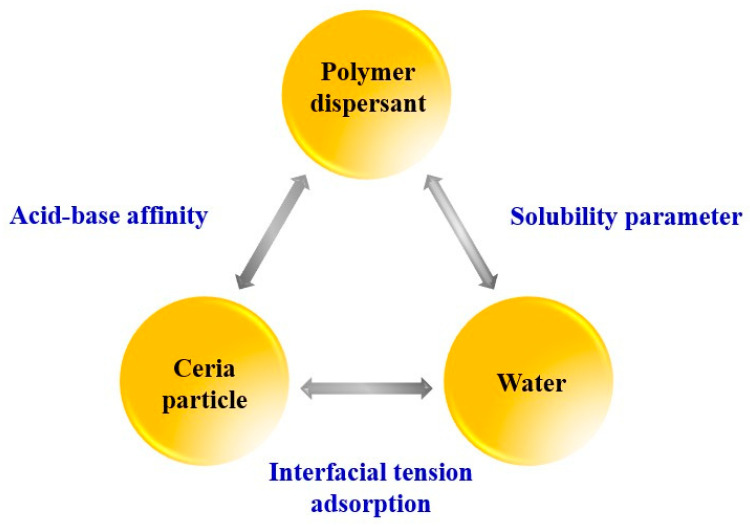
Three components in ceria particle as abrasive for CMP slurry [23].

Furthermore, achieving a balance of three components such as ceramic particles, water, and polymer dispersants is crucial for the dispersion of colloidal slurry. Specifically, the dispersibility of ceramic particles within the slurry is significantly influenced by the concentration of polymer dispersants, and variations in polymer dispersant content can lead to particle agglomeration. Therefore, it is highly important to maintain a balance among the three components of ceramic particles, polymer dispersants, and water at the composition in the slurry as illustrated in Figure 2 [16,17,18].

To achieve the high dispersed ceria slurry, there are many research to manufacture slurry particles with small, spherical, and uniformly monodisperse sizes with polymer dispersant as additives for CMP process. Huang’s group reported that size of ca. 70~100 nm at solid content of polymer nanoparticle with 1 wt% provide sufficient polishing RR and good total defect counts within the required targets, as well as good. In addition ceria-based abrasives prepared by nearly monodisperse near-spherical precursors show better uniformity and higher dispersion [19,20]. The polymer dispersants are generally adapted to enhance the dispersibility of ceria particles and stabilize their size distribution. The dispersibility of particles depends on the concentration of the polymer dispersant, which can also affect particle aggregation in the slurry. Feng and Zhao investigated metal oxide (Cr_2_O_3_) gel abrasive tools using polyacrylamide and polyimide resin as dispersants, examining the effects of dispersant concentration, slurry pH on slurry viscosity [21]. To enhance the abrasive dispersant stability in the CMP slurry, Park’s group involved to increase the polishing rate using ZrO_2_ as abrasive dispersant stability [22]. Furthermore, the stability tests were performed the ceria nanoparticle behavior based on dispersant polymer applied with deforming additives. The results indicated that the addition deforming polymer to the ceria slurry providing excellent colloidal stability in a previous study [23]. Therefore, the polymer dispersant establishes a balance among ceria, water, and the polymer dispersant present in the slurry, and it is essential that the composition of ceria, polymer dispersant, and water in the slurry is balanced (refer to Figure 2) [23,24].

**Figure 3:**
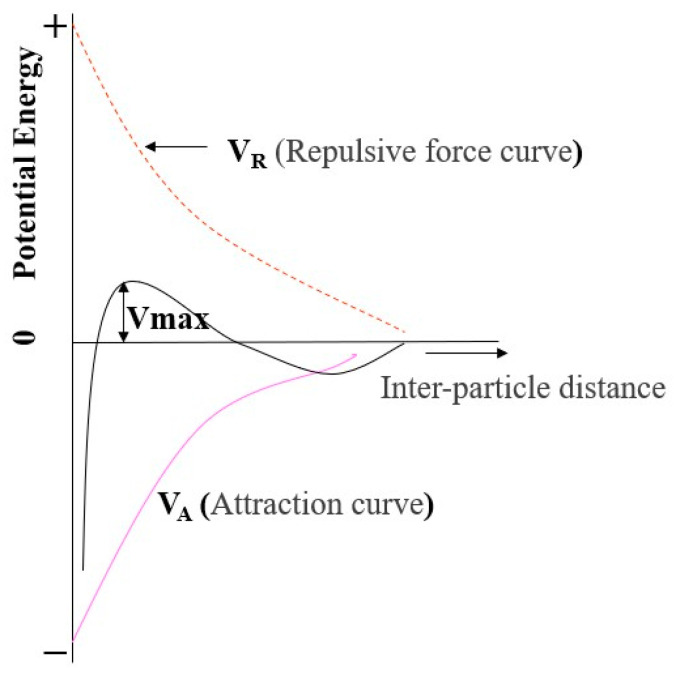
Schematic interaction energy vs. distance profiles of DLVO interaction. The attractive van der Waals and the repulsive electrostatic potentials form the total interaction energy [23].

According to DLVO theory, an optimized concentration of dispersant controlled electrostatic double-layer interactions, preventing close contact between ceria nanoparticles and maintaining dispersion, thereby reducing aggregation in Figure 3. As particle aggregation decreases, the problem of scratches during the CMP process is minimized, enabling more effective surface treatment and potentially enhancing polishing rates and selectivity [23,25].

Furthermore, adding a dispersant to the ceria slurry offers advantages by reducing issues such as non-uniformity, scratches, and defects that occur when the surface of the ceria particles carries a positive. Some groups have studied the preparation of ceria slurry with amino acid of ionic surfactant as additives [26].

Dandu Veera and Boumahraz researchers investigated the impact of RR using piperazine and imidazole [27]. Consequently, the ceria particles are affected by the dispersibility and agglomeration depending on the concentration of additives as polymer dispersants in the slurry. Therefore, the suitable selection of polymer dispersant materials can ultimately influence the performance of the RR. Therefore, selecting the appropriate polymer dispersants is important for improving the RR value in the CMP process.

**Figure 4:**
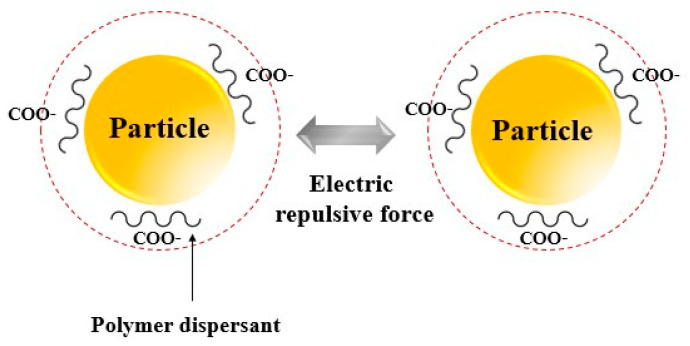
Illustration of dispersion mechanism using polymer dispersant.

Figure 4 illustrates that the dispersion mechanism of polymer dispersants involves the interaction between cohesive forces and electrostatic repulsive forces. The cohesive forces refer to the attractive interactions between ceria particles, and the dispersant weakens these forces, thereby preventing particle agglomeration. The polymer dispersant adsorbs onto the surfaces of the ceria particles, controlling the distance and interactions between them, which promotes better dispersion. Furthermore, the polymer dispersant enhances electrostatic repulsive forces by imparting similar charges onto the ceria particle surfaces, caused by the particles to repel each other. This electrostatic repulsion prevents the particles from aggregation, thereby ensuring a uniform dispersion in ceria slurry.

In previous study, stability tests were performed the ceria nanoparticle behavior based on styrene maleic acrylate (SMA) dispersant polymer applied with 3-types of deforming additives. The results indicated that the addition G-336 deforming polymer to the ceria slurry providing excellent colloidal stability [23]. This demonstrates that a stable adsorption interaction between ceria particles and polymer dispersants is of great importance. The proper selection of dispersants and abrasives in CMP slurry is essential, as it prevents the agglomeration of ceria particles, enhances slurry stability, and improves both the removal rate and selectivity. Thus, the choice of dispersants and abrasives is a critical factor in the CMP process.

To our best knowledge, polymer dispersants were prepared with different contents using zinc salt of ethylene acrylic acid (EAA) copolymer and applied to ceria slurry to minimize the issue of particle aggregation commonly observed in CMP process. Especially, the adapted zinc salt of the EAA copolymer is expected to improve the thermal stability and rheological properties of the interlayer between ceria nanoparticles [28]. In addition, stability tests were conducted to evaluate the effect of temperature changes on the polymer dispersant’s impact on the dispersion stability of ceria particles. These tests were performed at temperature conditions of 25 °C (opened and closed systems), 4 °C, and 60 °C, respectively.

## 2. Experimental

### 2.1. Materials

#### Preparation of Ceria Slurry with Calcined Ceria Nanoparticle

Cerium carbonate hydrate (Ce_2_(CO_3_) _3_)∙6H_2_O (99.9% by Sigma-Aldrich, St. Louis, America)powder was calcined at 800 °C to obtain cerium oxide (CeO_2_) powder for use as abrasive particles in polishing test. To prepare the ceria slurry, DI water (aquapuri 5 series by YOUNG IN SCIENTIFIC, Co., Ltd. Seoul, Republic of Korea) and an acrylic acid-based dispersant with various content 5 wt%, 6 wt%, 7 wt% (denoted by D5, D6 and D7)., respectively using zinc salt of ethylene acrylic acid (EAA) copolymer (Vanderbilt Minerals, LLC, New York, USA) were used comparison with commercial dispersion material

The ceria slurry was prepared with a fixed ceria concentration of 30%, dispersant concentrations of 5 wt%, 6 wt%, and 7 wt%, and DI water adjusted to complete a total volume of 100 wt% using a Basket-mill equipment. (Tedi, JS Basket-mill Mill, Daejeon, Republic of Korea). During dispersion, beads with a size of 0.2 mm were utilized with a bead filling ratio set at 60%. Additionally, milling process was conducted at 1500 rpm for 3 h. The obtained slurry was then diluted to achieve solid content with fixed amount. All prepared smples were performed comparions with physico-chemical properties of commercial dispersant ceria slurry.

### 2.2. Characterization

#### 2.2.1. Analyze of Ceria Slurry

To evaluate the physico-chemical properties and stability of the prepared and commercial slurry, a pH meter (Thermo Fisher Scientific, Orionstars A215, Waltham, MA, USA), conductivity meter (Thermo Fisher Scientific, Orionstars A215, Waltham, MA, USA), and viscometer (Brookfield, DVNext Cone/Plate Rheometer, Boston, MA, USA) were employed. A zeta potential analysis (Microtrac, Nanotrac Wave II, Osaka, Japan) was conducted to assess the dispersion of slurry particles, and dynamic light scattering (DLS, ELS-2000, Otsuka Electronics, Osaka, Japan) was used to measure particle size distribution (PSD). Additionally, attenuated total internal reflectance-infra red (ATR-IR) spectroscopy (Thermo Fisher Scientific, Nicolet Summit X, Waltham, MA, USA) was conducted for the component analysis of the prepared and commercial ceria slurry. To observe the shape and size of the slurry particles, a field emission scanning electron microscope (FE-SEM, JEOL-7800F, JEOL Ltd., Tokyo, Japan) was performed. Furthermore, to analyze the adsorption and orientation of polymer dispersants on the ceria surface, a transmission electron microscope (TEM, NEOARM JEM-ARM 200F, JEOL Ltd., Tokyo, Japan) was employed.

#### 2.2.2. Stability Test of Ceria Slurry

The prepared slurry with according to concentrations was evaluated to stability testing under extreme conditions such as opened and closed at 25 °C (opened and closed condition), 4 °C and 60 °C, respectively, for three months compared to commercial slurry. Measurements of DLS, pH, conductivity, and viscosity were performed per 1 week. Analyses of contact angle, zeta potential, DLS, pH, conductivity, and viscosity was conducted to assess changes in physical properties between the initial and after three months of ceria slurry.

### 2.3. Evaluation of Removal Rate (RR) and Uniformity

All ceria slurry was carried out polishing tests using AP-300 equipment. A pads (IC1010 TM A6 PAD) were set to rotate at a speed of 93 rpm under a downward load, and the slurry flow rate was maintained at 180 mL/min. A uniform polishing time of 60 s was set, and conditioning was performed for 10 min using a conditioner. The wafers used included PETEOS with diameter of 200 mm. The experiment was conducted at a pressure of 9 psi, and the pH of slurry was in the range of 7 to 8. The uniformity of ceria slurry is measured using a thickness gauge (ST2000D LXn, SJT Ltd., Daejeon, Republic of Korea) by applying the slurry to a substrate and taking measurements at multiple points across the surface. In addition, for the purpose of comparison, the pad before polishing was employed as a control group to evaluate the polishing rate for each slurry.

## 3. Results and Discussion

### 3.1. The Solid Content of Ceria Slurry

The ceria slurry with acrylic acid-based dispersant adapting zinc salt of ethylene acrylic acid (EAA) copolymer was prepared using calcined ceria (CeO_2_) particles as abrasive for CMP process. To confirm the pure content of the dispersant, solid content analysis of the ceria slurry was examined.

**Figure 5:**
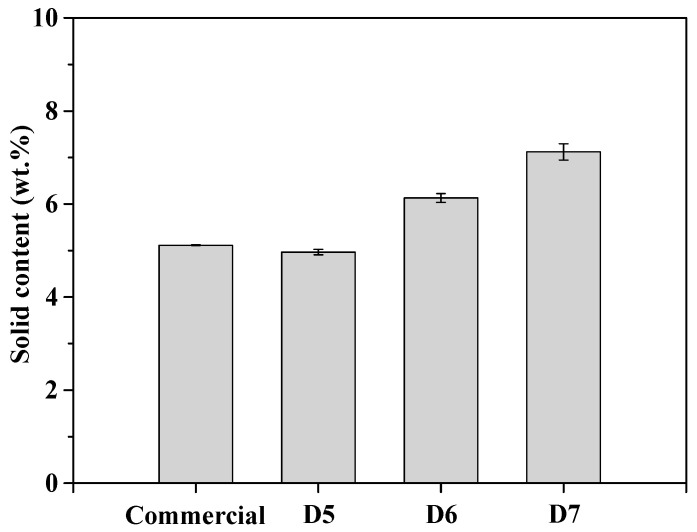
Solid contents of prepared ceria slurries according to concentration compared to commercial slurry.

Figure 5 shows the solid content of the prepared ceria slurry comparison with commercial ceria slurry. All samples were performed by placing 1 g of each slurry in a petri dish and then drying process at 60 °C in oven, followed by measuring the weight change before and after drying. The solid content of commercial polymer dispersant and prepared polymer dispersants were measured at 5.1210 wt.%, 4.9531 wt.%, 6.1489 wt.%, 7.1379 wt.%, respectively. The prepared samples with 3-types of polymer dispersant materials were denoted by D5, D6, and D7, respectively.

### 3.2. Physico-Chemical Properties of Commercial and Prepared Slurry at Initial Condition

To confirm the physico-chemical properties of the commercial and prepared ceria slurry with varying polymer dispersant contents, particle size distribution (PSD), optical microscope analysis (OM), contact angle (CA), pH and conductivity, ATR-IR and viscosity analysis, were performed.

**Figure 6:**
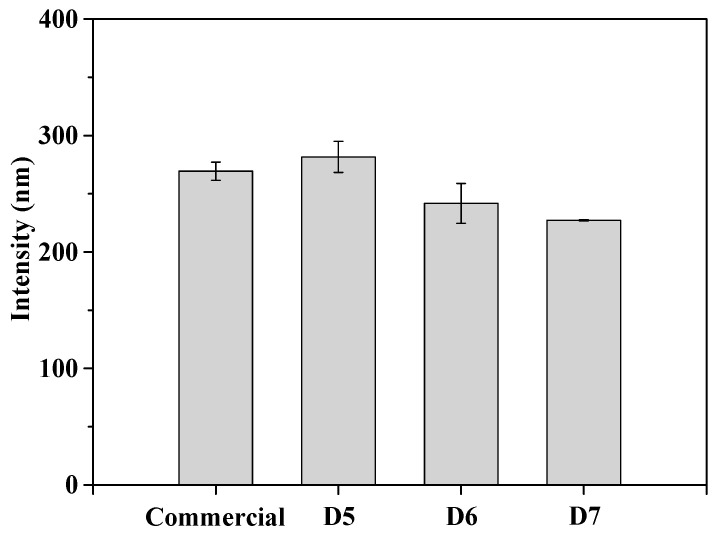
Particle size of prepared ceria slurries according to concentration compared to commercial slurry.

Figure 6 presents the particle size distribution (PSD) and D90 values of the particle sizes for both the commercial slurry and the prepared slurry, measured by DLS equipment. The particle size measured at 281.6 nm, 251.8 nm, 227.2 nm for D5, D6, D7, respectively. The results indicate that as the polymer dispersant content increases, the particle size of the ceria in the slurry decreases. This is considered to be due to better adsorption of the polymer dispersant onto the surface of ceria particle, leading to an increase in electrostatic repulsion between particles, which in turn decreases the particle size. Table 1 summarizes the results of particle size with the average values and error rates for commercial and prepared slurry.

**Figure 7:**
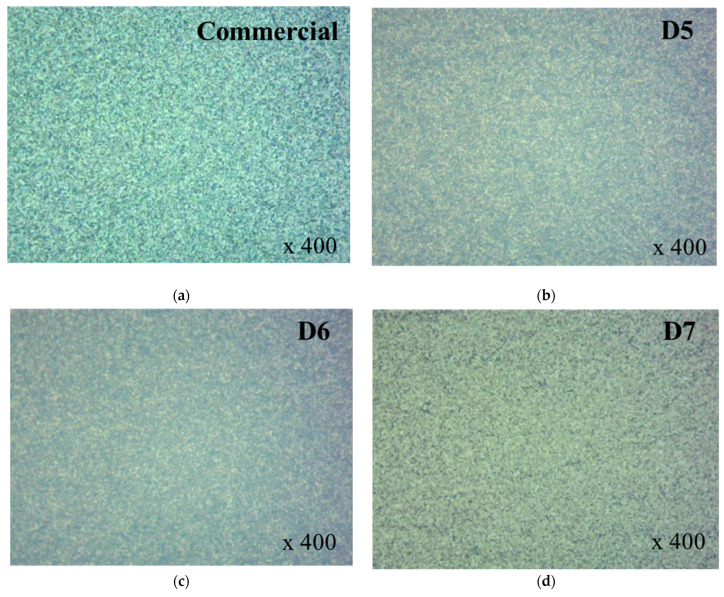
Optical microscope images of commercial and prepared ceria slurries. (**a**) Commercial ceria slurry; (**b**–**d**) Prepared ceria slurries with 5, 6, 7 wt% content, respectively, as noted D5, D6 and D7.

Optical Microscope (OM) analysis was conducted to evaluate the initial dispersion and agglomeration behavior of ceria particles in both commercial and prepared slurries with different contents in Figure 7. In addition, the D7 sample demonstrated a particle behavior similar to that of the commercial slurry. It was confirmed through the OM results that none of the all sample of slurry exhibited particle agglomeration, indicating uniform dispersion. The D5 and D6 samples demonstrated similar particle behavior, while D7 showed particle behavior comparable to that of the commercial sample. These differences are expected to impact the physicochemical properties of the slurry.

**Figure 8:**
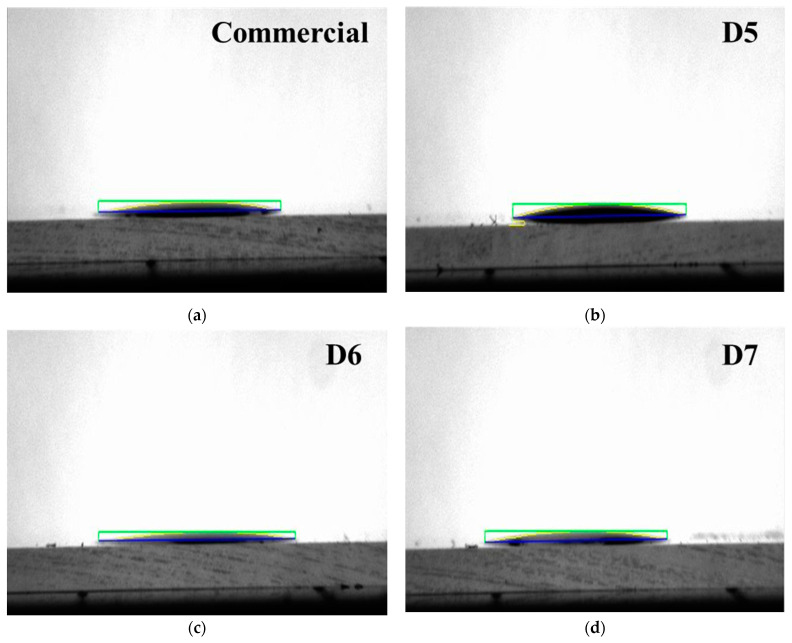
Contact angle of commercial and prepared ceria slurries. Images (**a**–**d**) corresponds to the same field at a same magnification on initial condition. (**a**) Commercial ceria slurry; (**b**–**d**) Prepared ceria slurry with 5, 6, 7 wt% content, respectively as noted D5, D6 and D7.

To evaluate the degree of hydrophilicity according to the polymer dispersant content, the contact angles were measured as shown in Figure 8. Meanwhile, after dropping a ceria slurry droplet onto the specimen, a camera was used to capture images when the liquid droplet made contact with the surface of the specimen. The captured images were then automatically analyzed to calculate the contact angle using an image analysis program. The values of contact angle were measured at 4.77°, 7.04°, 5.90° and 4.32°, respectively with commercial and prepared ceria slurry. Especially, it was found to be approximately 10% more hydrophilic of the D7 sample compared to the commercial slurry. This result is likely due to the increase in carboxyl groups (-COOH) contained in the polymer dispersant, which increases along with the polymer dispersant content, leading to more hydrophilic behavior. It is expected to also influence the stability of the ceria slurry.

The pH and conductivity measurement results for the prepared samples indicates comparison with commercial slurry in Table 2. The pH measurement showed that the commercial slurry tested a pH of 8.48, while the prepared slurry measured 9.46, 9.51, and 9.49, respectively, with no significant differences based on the polymer dispersant content. The higher pH of the prepared slurry is likely due to the zinc salt of ethylene acrylic acid (EAA) copolymer of the dispersant. The conductivity showed a tendency of increasing values as the polymer dispersant content increased at 423.2 μS/cm, 508.4 μS/cm, and 701.8 μS/cm in case of D5, D6 and D7 sample. This suggests that the ion components in the polymer dispersant and the superior adsorption of the polymer dispersant on the ceria surface contributed to the increase in conductivity. It exhibited approximately 17% higher conductivity than the commercial slurry in the D7 sample.

**Figure 9:**
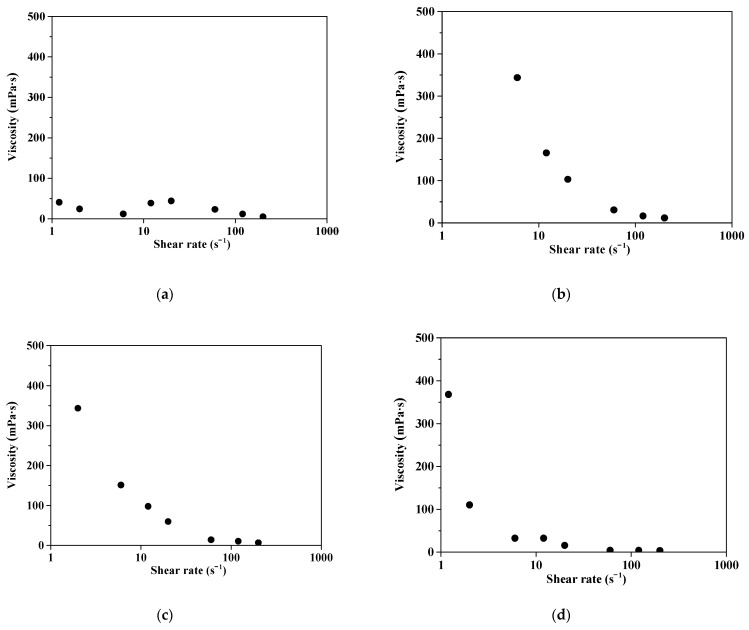
Measurements of viscosity vs. shear rate: (**a**) commercial ceria slurry; (**b**–**d**) Prepared ceria slurry with 5, 6, 7 wt% content, respectively as noted D5, D6 and D7.

Figure 9 shows the measurements of viscosity vs. shear stress for the all ceria slurry samples. The viscosity of the commercial ceria slurry decreases as the shear rate increases, indicating typical shear-thinning behavior. The results of viscosity versus shear rate measurements indicate that the commercial dispersant exhibits Newtonian behavior, while the viscosity of the prepared dispersant decreases as its content increases. The viscosity of the commercial dispersant generally remains low as the shear rate increases. This indicates that the agglomeration of ceria particles is minimized, maintaining a stable slurry viscosity. The D5 slurry shows unstable viscosity with particle agglomeration or recombination occurring as the shear rate increases, resulting in a high viscosity around a shear rate of 8. In contrast, the D7 slurry exhibits a low viscosity even at lower shear rates. This suggests that as the dispersant content increases, the slurry viscosity becomes more stable. This suggests that an increased dispersant content in the CMP slurry introduces more carboxyl groups (-COOH), transitioning the slurry’s behavior from thixotropic, characterized by viscoelastic stress, to Newtonian behavior, where the viscosity is linear and independent of shear rate.

**Figure 10:**
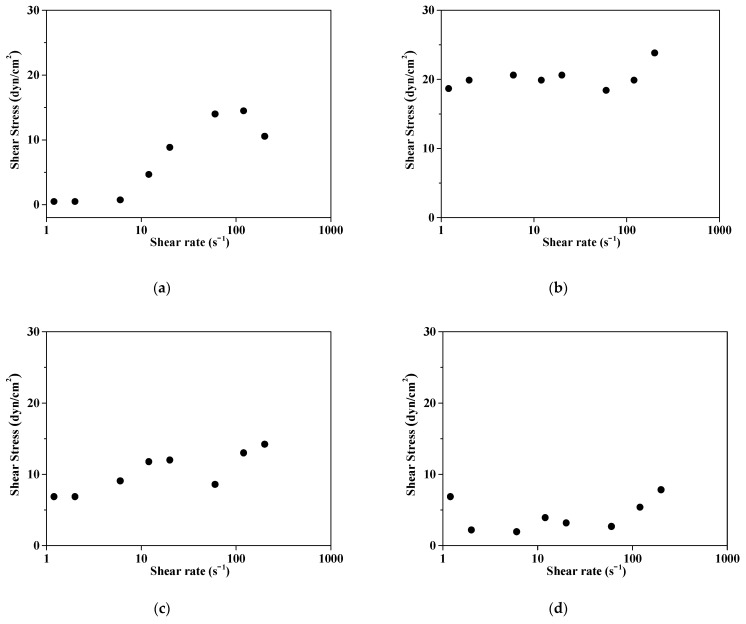
Measurements of shear stress vs. shear rate: (**a**) commercial ceria slurry; (**b**–**d**) Prepared ceria slurry with 5, 6, 7 wt% content, respectively as noted D5, D6 and D7.

Figure 10 presents the shear stress for the commercial slurry and prepared slurries. Shear stress represents the force required to separate particles, and higher values indicate stronger forces and lower dispersibility. Shear Stress is the shear force acting on the fluid, which resists the movement of particles or molecules as the fluid flows. Shear Rate refers to the rate at which particles move within the fluid, which is related to the fluid’s shear velocity. For the commercial dispersant, as the Shear Rate increases, the Shear Stress tends to increase around a value of approximately 10 s^−1^, indicating an increase in viscosity or an unstable ceria slurry. The shear stress vs. shear rate showed that the D5 ceria slurry had a shear stress value below 30, while D6 and D7 were measured below 20. The D7 sample illustrates the lowest shear stress, indicating that as the polymer dispersant content increases, the stress falls below 20, leading to more stable behavior. On the other hand, as the dispersant content increases, the Shear Stress tends to decrease with increasing Shear Rate, which indicates that the slurry viscosity remains stable, resulting in stable flow characteristics.

**Figure 11:**
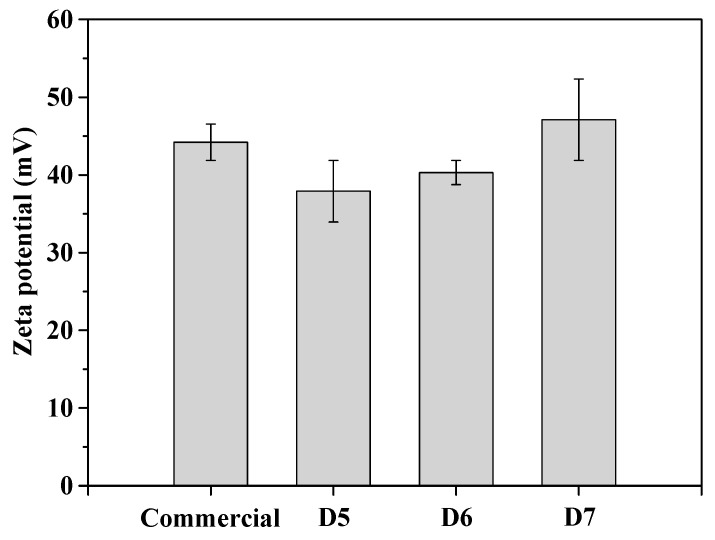
Comparison of zeta potential of prepared ceria slurries with polymer dispersant using different contents.

Figure 11 analyze the zeta potential of the electrostatic potential of the electric double layer surrounding ceria nanoparticles in a slurry solution. It showed that zeta potential indicates 49.2 mV, 42.9 mV, 45.3 mV and 52.1 mV in case of commercial, D5, D6 and D7 slurry. This indicates that the zeta potential increases as the polymer dispersant content increases, suggesting higher dispersibility with higher polymer dispersant concentrations.

**Figure 12:**
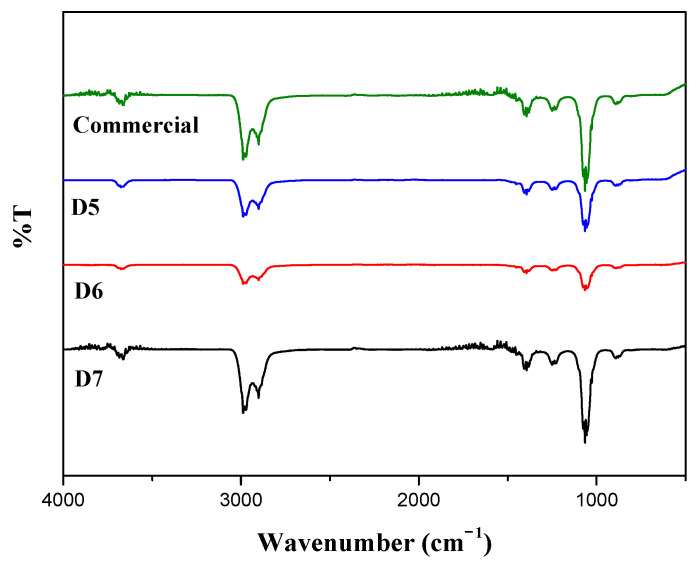
Infrared spectra of prepared ceria slurries compared to commercial slurry.

ATR-IR analysis was conducted to examine the IR spectrum of prepared ceria slurry. Based on the molecular characteristics that absorb infrared radiation in the range of 400 to 4000 cm^−1^ in Figure 12. It was observed that the prepared slurry exhibited peaks at similar tendency compared to the commercial slurry. A peak associated with the C-O bond of the carboxyl group is observed around 1100 cm^−1^, and an absorption peak corresponding to the C-H bond of hydrocarbons is identified about 2800 cm^−1^. The prepared all samples show IR peak results similar to those of the commercial polymer dispersant, while the concentration of D series increases, the intensity of the peaks with sharpness increases. Especially, presence of zinc salt of ethylene acrylic acid (EAA) copolymer was confirmed. It is expected that the stretching vibration peak of the C-H bond at 2900 cm^−1^ is due to the interaction between zinc and acrylic acid [29]. Additionally, as the polymer dispersant content in the prepared slurry increased, the size of the detected peaks grew, indicating an increase in the amount of zinc salt of ethylene acrylic acid copolymer.

**Figure 13:**
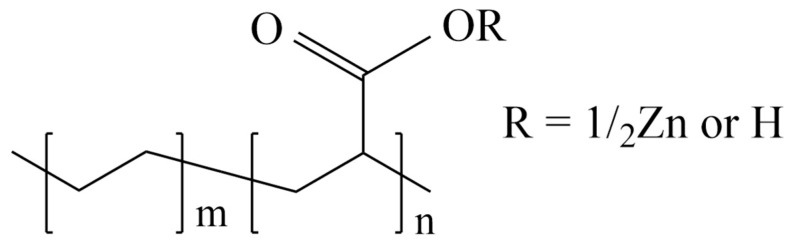
Chemical formula structure of zinc salt of ethylene acrylic acid (EAA) copolymer.

Figure 13 shows the chemical structure of the zinc salt of ethylene acrylic acid (EAA) copolymer. This copolymer is expected to help prevent the agglomeration of ceria particles in the ceria slurry, maintaining dispersion stability. By inhibiting particle aggregation, the zinc salt of EAA copolymer plays a crucial role in keeping the ceria slurry uniformly distributed, which is essential for consistent processing and performance across various applications [28].

**Figure 14:**
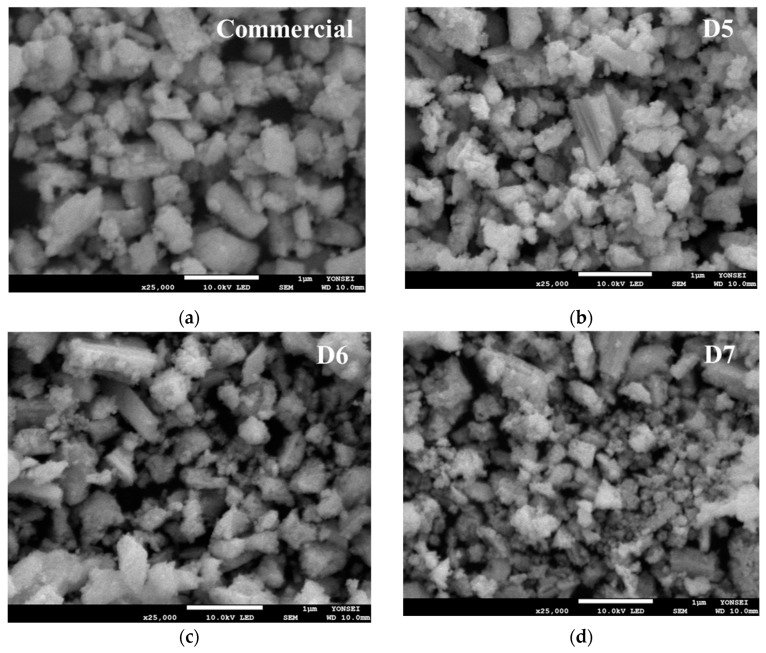
SEM images of prepared ceria nanoparticle in slurry according to the polymer dispersant contents comparison with commercial ceria nanoparticle.: (**a**) Commercial ceria slurry; (**b**) Ceria nanoparticle in D5 sample; (**c**) Ceria nanoparticle in D6 sample; (**d**) Ceria nanoparticle in D7 sample.

The surface morphologies of ceria nanoparticles with varying polymer dispersant contents were characterized using SEM analysis (Figure 14). The SEM image of the ceria nanoparticles reveal that the particle size of the D5 and D6 samples is confirmed to be below approximately 1 µm similar to ceria particle of commercial slurry. However, the size of ceria particle is significantly reduced to a range of several tens to hundreds of nm in the case of the D7 ceria slurry, likely due to the effect of the polymer dispersant.

**Figure 15:**
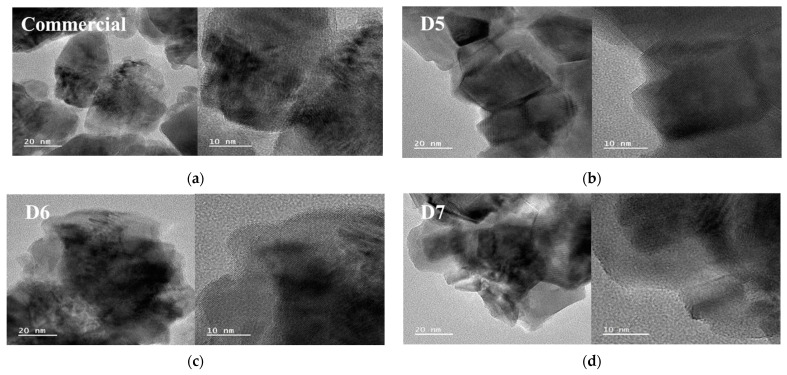
TEM images of prepared ceria nanoparticle in slurry according to the polymer dispersant contents comparison with commercial ceria nanoparticle.: (**a**) Commercial ceria slurry; (**b**) Ceria nanoparticle in D5 sample; (**c**) Ceria nanoparticle in D6 sample; (**d**) Ceria nanoparticle in D7 sample.

Figure 15 shows the TEM images of commercial slurry and 3-types of prepared slurry. It can be observed that as the amount of the polymer dispersant increases, the size of the ceria particles decreases. A uniform orientation of the ceria particles is observed with additive as polymer dispersant being most thickly adsorbed on the prepared D7 ceria slurry. This indicates that as the concentration of the polymer dispersant increases, the polymer dispersant can stably adsorb onto the surface of ceria particles. In particular, it is expected that the polymer adsorbs with a stable thickness of approximately 5 nm, which can enhance the stability of the ceria particles for the D7 sample.

### 3.3. Long Term Stability Properties of Commercial and Prepared Slurry

Generally, the ceria nanoparticles tend to aggregate easily due to their high surface energy, it is difficult for them to remain uniformly dispersed in DI water for extended periods [30]. This can lead to agglomeration or scratches on the wafer during the CMP process, which results in a reduced material removal rate (RR) performance.

Therefore, a long-term stability of commercial slurry and prepared ceria slurry with varying polymer dispersant contents was evaluated in this study. All slurry was characterized by measuring DLS, pH, conductivity, and viscosity, respectively at 25 °C (opened/closed condition), 4 °C and 60 °C for three months.

**Figure 16:**
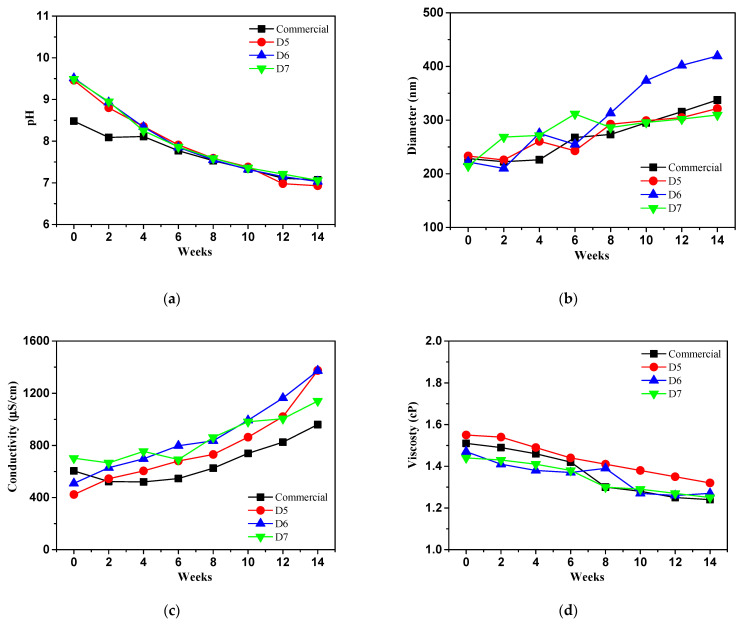
Long term physico-chemical stability test of prepared ceria slurry comparison with commercial ceria slurry at 25 °C (opened condition) for three months.: (**a**) pH; (**b**) particle size; (**c**) conductivity; (**d**) viscosity.

Figure 16 indicates a long term physico-chemical stability test of prepared ceria slurry those of commercial ceria slurry as reference in which pH, particle size, conductivity and viscosity at 25 °C (opened condition) for three months. The prepared samples showed a decreasing tendency of pH in range of 6.93 to 7.06 after finished stability test. Meanwhile, the pH values of the D7 ceria slurry were similar to commercial ceria slurry, measuring 7.06 and 7.07, respectively.

DLS analysis was evaluated to measure the size of ceria particles in ceria slurry at 25 °C (opened condition) after three months. All samples showed an increase with an irregular shape in particle size. Especially, the particle size of the commercial ceria slurry increased from 228 nm to 337 nm with ratio of 32%, while the D6 and D7 samples exhibited relatively smaller increases in size, with 27% and 31%, respectively.

Figure 16c shows the conductivity of the prepared ceria slurry, where the conductivity of all samples remained constant up to 6 weeks but began to increase after 8 weeks. D5 and D6 showed significant increases of 69% and 62%, respectively, while D7 sample displayed with ratio of 31% a similar increasing tendency to the commercial ceria slurry with ratio of 32%. This data aligns with the DLS results, where the particle size of the ceria in D7 showed a similar increase to that of the commercial ceria slurry.

All samples showed maintaining viscosity values, and the viscosities of prepared ceria slurry such as D5, D6, and D7 exhibited changes of 0.23 cP, 0.2 cP, and 0.19 cP, respectively. Compared to the commercial ceria slurry, a lower rate of decrease was observed in Figure 16d.

**Figure 17:**
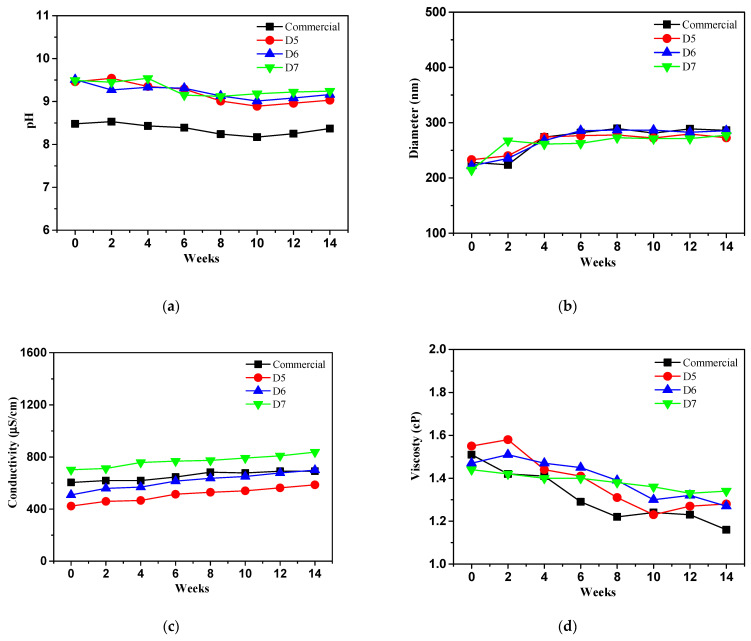
Long term physico-chemical stability test of prepared ceria slurry comparison with commercial ceria slurry at 25 °C (closed condition) for three months.: (**a**) pH; (**b**) particle size; (**c**) conductivity; (**d**) viscosity.

The commercial slurry and prepared slurry were evaluated pH, particle size, conductivity and viscosity at 25 °C (closed condition) for three months as reference condition (Figure 17). The pH of the commercial ceria slurry decreased from 8.48 to 8.37, while the prepared ceria slurry changed from 9.46 to 9.03, 951 to 9.16, and 9.49 to 9.24, respectively in case of D5, D6 and D7 samples. The prepared ceria slurry showed more alkaline conditions compared to the commercial ceria slurry. It indicates that more ceria nanoparticles will be adsorbed on surface of silicon oxide [31]. Therefore, it will have expected to obtain a smoother and more planer oxide surface area during CMP process.

DLS analysis was performed to evaluate the particle size of ceria slurry for all samples. The particle size of the commercial ceria slurry increased by 8%, from 228 nm to 246 nm, while D5 increased by 5%, from 232 nm to 242 nm, D6 increased by 14%, from 221 nm to 255 nm, and D7 increased by 6%, from 214 nm to 227 nm. It was observed that the ceria particles of the prepared slurry increased less in size compared to the commercial slurry except for the D6 sample.

Conductivity analysis was performed to evaluate the conductivity of ceria particle for all samples according to weeks. All samples showed an increasing conductivity by approximately 13%, from 604 µS/cm to 690 µS/cm, 28% from 423 µS/cm to 586 µS/cm, 28%, from 508 µS/cm to 699 µS/cm, and 17%, from 701 µS/cm to 837 µS/cm, in case of commercial, D5, D6 and D7 samples. Although the size of ceria particles lead to increase with higher conductivity it was confirmed that the growth of ceria particles, except for the D6 sample, was not significantly larger compared to commercial slurry at 25 °C (closed condition). It is likely that the conductivity of the all ceria slurry was not excessively high, and therefore did not affect the aggregation of the ceria particles.

A commercial ceria slurry showed a changed value of 0.25 cP decreasing from 1.41 cP to 1.16 Cp, while the prepared ceria slurry decreased 0.34 cP from 1.62 cP to 1.28 cP, 0.20 cP from 1.47 cP to 1.27 cP, 0.10 cP from 1.44 cP to 1.34 cP, respectively in D5 to D7 samples in viscosity analysis. D5 sample decreased significantly a change of 0.34 cP. The prepared ceria slurry was lower viscosity compared to commercial ceria slurry, and it was confirmed that the D7 sample exhibited the least changes value. This suggests that the ceria particles in the D7 ceria slurry are likely less agglomerated are stably dispersed.

**Figure 18:**
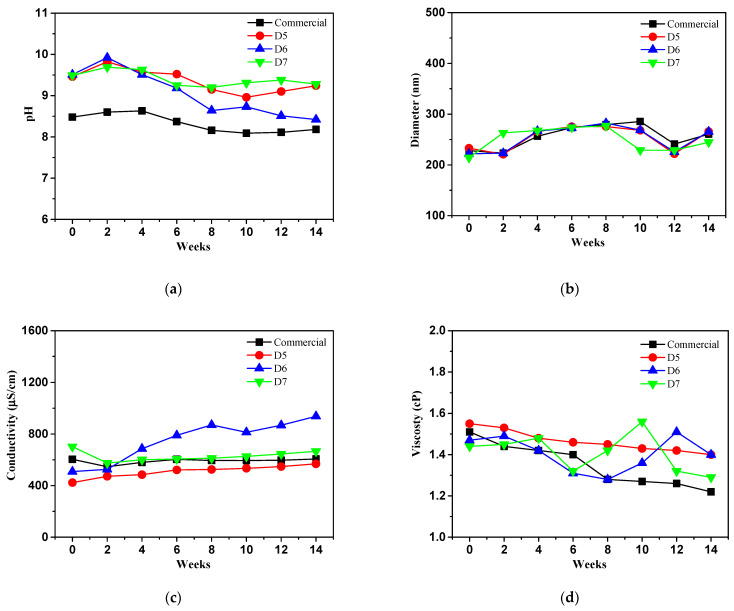
Long term physico-chemical stability test of prepared ceria slurry comparison with commercial ceria slurry at 4 °C for three months: (**a**) pH; (**b**) particle size; (**c**) conductivity; (**d**) viscosity.

Figure 18 shows a long term physico-chemical stability test of prepared ceria slurry in which pH, particle size, conductivity and viscosity at 4 °C for three months.

The pH of reduced value of the commercial ceria slurry, D5 and D7 samples slightly decreased by 0.30, 0.22 and 0.21, respectively, except D6 sample with 1.09 as shown in Figure 18a. This is likely due to the significant inhibition of chemical reactions and the movement of materials in the ceria slurry under low temperature condition. In other words, the mobility and reactivity of ions are greatly reduced, resulting in minimal pH changes. The prepared D5 and D7 samples still maintained an alkaline media condition (≤9.2), except for the D6 sample, while the commercial ceria slurry has a neutral media environment.

The particle size of all ceria slurry shows a tendency similar to that of the pH changes in Figure 18b. The sizes increased from 228 nm to 260 nm, from 232 nm to 265 nm, from 221 nm to 265 nm, from 214 nm to 244 nm in cases of commercial and prepared D5, D6 and D7 samples, respectively. The ceria particle sizes of the commercial slurry and the prepared D7 sample increased by approximately 12%, showing similar values.

Figure 18c shows the conductivity values of all ceria slurry under 4 °C conditions. Meanwhile, the conductivity of the commercial ceria slurry remained constant, whereas the D5 and D6 samples showed increases of approximately 26% and 36%, respectively. However, the D7 sample exhibited a decrease in conductivity. It seems that as the temperature decreases, ion mobility significantly decreases, and if ions cannot move freely, the electrical conductivity is reduced. Additionally, chemical reactions within the slurry may be inhibited, which is likely the reason for these data.

Furthermore, the viscosity of commercial and prepared ceria slurry showed such as 0.15 cP, 0.09 cP, and 0.16 cP, respectively. Compared to the commercial ceria slurry with 0.29 cP, a lower rate of decrease was observed in Figure 18d. The decrease in viscosity seems to be due to weakened interactions between particles, allowing the particles in the ceria slurry to move more freely at 4 °C.

**Figure 19:**
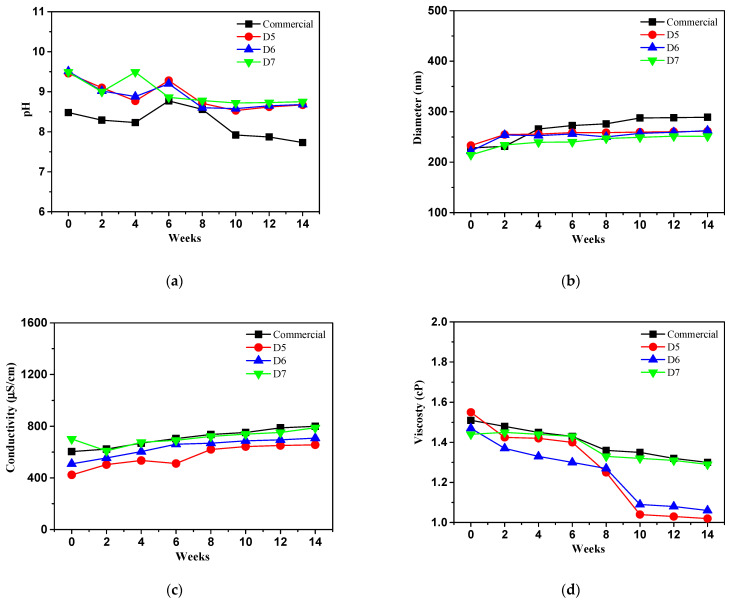
Long term physico-chemical stability test of prepared ceria slurry comparison with commercial ceria slurry at 60 °C for three months.: (**a**) pH; (**b**) particle size; (**c**) conductivity; (**d**) viscosity.

The stability of pH, particle size, conductivity, and viscosity for commercial ceria slurry and prepared ceria slurry were evaluated at 60 °C for three months, as shown in Figure 19. The pH values of all samples decreased from 8.48 to 7.73, 9.46 to 8.67, 9.51 to 8.68, and 9.49 to 8.75 with range of 7 to 8 for commercial, D5, D6 and D7 samples, respectively. It appears that the pH decrease may be due to the gradual degradation of the polymer dispersant at the high temperature of 60 °C. Furthermore, the pH reduction was found to be similar in both the commercial ceria slurry and the D7 sample slurry, with Δ pH decreases of 0.75 and 0.74, respectively. Meanwhile, the ceria nanoparticle size of the commercial ceria slurry increased by 21%, from 228 nm to 289 nm, whereas the ceria particle sizes of the prepared ceria slurry increased by 10%, 15%, and 14% from 232 nm to 261 nm, 221 nm to 262 nm, and 214 nm to 251 nm, respectively for D5, D6, and D7 samples.

The growth of ceria nanoparticle size appears to result from a reduction in repulsive forces between the ceria particles, such as electrostatic or steric repulsion, which increases the probability of particle agglomeration at 60 °C comparison with the conditions at 25 °C and 4 °C. In addition, the higher rate of size growth may be attributed to changes in the surface chemistry of the ceria nanoparticles for the commercial ceria slurry, potentially caused by the removal of the polymer dispersant that stabilizes the nanoparticles.

To evaluate the stability of all samples at high temperatures (60 °C), conductivity was measured by conductivity meter after three months. The conductivity of the commercial ceria slurry increased by 24.5%, from 604.1 µS/cm to 800.2 µS/cm, while the prepared ceria slurry increased by 35.5% from 423.2 µS/cm to 656.4 µS/cm, 28.2% form 508.4 µS/cm to 708.1 µS/cm, and 11% from 701.8 µS/cm to 788.7 µS/cm respectively, for D5, D6, and D7 samples. The conductivity with change of the prepared D7 ceria slurry was measured to be more than two times smaller compared to the commercial ceria slurry. This suggests that the ceria colloidal stability of the prepared D7 sample is maintained better compared to the commercial ceria and D5, D6 slurry, decreasing the risk of agglomeration or sedimentation. As a result, it can be expected that the prepared D7 ceria slurry will exhibit minimal degradation at high temperatures compared to the other samples.

The viscosity change rates of the prepared D5, D6, and D7 ceria slurry were measured at 0.39 cP, 0.23 cP, and 0.03 cP respectively, after being stored at 60 °C for three months. The prepared D7 ceria slurry showed a difference of about seven times compared to the viscosity lower change rate with 0.21 cP of than the commercial ceria slurry. Considering the ceria size and conductivity result, it appears that the prepared D7 ceria slurry maintains the most stable state at high temperatures.

### 3.4. Removal Rate (RR) Evaluation of Prepared Ceria Slurry

Polishing experiments of commercial and prepared ceria slurries was conducted to evaluate the removal rate rate and uniformity of the ceria slurry.

**Figure 20:**
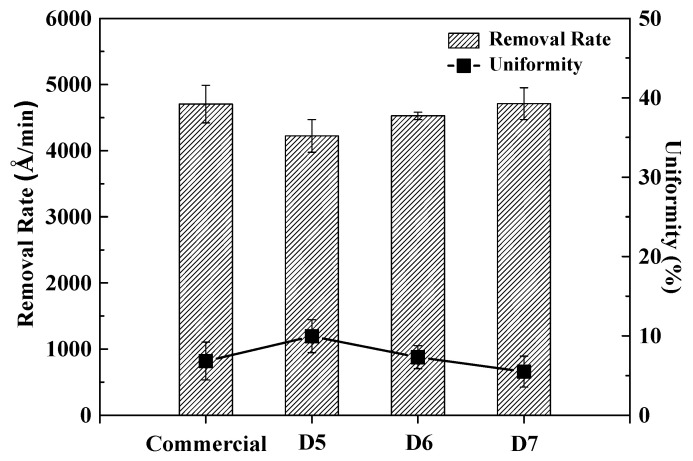
Removal rate and uniformity of commercial and prepared ceria slurry.

Figure 20 presented the RR and uniformity results for commercial and prepared ceria slurry with according to the polymer dispersant contents after PETEOS polishing evaluation. The preapred ceria slurry with according to contents of the polymer dispersant increased to 4224 Å/min, 4538 Å/min, and 4712 Å/min, while the commercial ceria slurry showed a of 4706 Å/min. The prepared D7 ceria slurry exhibited a slightly higher RR compared to the commercial ceria, which can be attributed to the fact that the ceria nanoparticles consist of smaller particle sizes than those in the commercial slurry. Additionally, the polymer dispersant is stably adsorbed onto the surface of the ceria particles in a thick layer, as shown in the TEM and SEM microscopy images.

The uniformity evaluation was conducted to determine whether the surface of all samples was evenly removed during the polishing process. The uniformity of the prepared ceria slurry based on polymer dispersant content was found to be 9.95%, 7.31%, and 5.48% respectivley, while the uniformity of the commercial ceria slurry was evaluated at 6.82%. This indicates that the dispersant content of D7 sample enhance interparticle repulsive forces, positively influencing the uniformity comparison with commercial slurry. which is slightly better than that of the D7 ceria slurry. This can be attributed to the fact that the increased polymer dispersant content allowed the ceria particles in the prepared ceria slurry to be more widely spaced apart, enhancing dispersion and reducing interactions between particles, thereby contributing to the formation of a uniform slurry. Additionally, polymer dispersants adsorb onto the ceria surfaces, providing charge and creating steric hindrance that prevents particle agglomeration. As a result, it is believed that increasing the polymer dispersant content can enhance the uniformity of the slurry.

## 4. Conclusions

In this study, prepared ceria slurry using polymer dispersant with zinc salt of ethylene acrylic acid (EAA) copolymer at different contents such as 5, 6 and 7 wt.% (denoted by D5, D6 and D7), respectively, to minimize the issue of particle aggregation commonly observed in CMP slurry. All samples characterized by pH, particle size, conductivity and viscosity to evaluate physico-chemical properties compared to commercial slurry initial and evaluated stability test at 25 °C (opened/closed condition), 4 °C and 60 °C for three months.

The prepared D7 ceria slurry exhibited a slightly higher removal rate (RR) and better uniformity compared to the commercial ceria slurry. This can be attributed to the smaller particle size of the ceria nanoparticles in the D7 slurry. Additionally, the increased polymer dispersant content facilitated better dispersion by decreasing particle interactions and enhancing a more uniform slurry formation in the ceria slurry

## Figures and Tables

**Table 1:** Particle size with the average values and error rates for commercial and prepared slurry.

PSD (D90, nm)	Commercial	D5	D6	D7
1	257.7 ± 11.7	290.7 ± 9.10	248.2 ± 3.60	227.4 ± 0.20
2	273.3 ± 3.90	261.6 ± 20.0	229.7 ± 22.1	226.4 ± 0.80
3	277.1 ± 7.70	292.4 ± 10.8	277.5 ± 25.7	227.9 ± 0.70
Average	269.4 ± 7.76	281.6 ± 13.3	251.8 ± 17.1	227.2 ± 0.57

**Table 2:** The pH and conductivity measurement results for the prepared samples.

Contents	Commercial	D5	D6	D7
pH	8.48	9.46	9.51	9.49
Conductivity (μS/cm)	604.1	423.2	508.4	701.8

## Data Availability

Data are available on request to the corresponding author.

## References

[1] Han X., Zhang S., Liu R., Wang F., Tan B., Zhao X., Zhao J., Shi Y. (2024). The effect of amino acid addition in CeO2-based slurry on SiO_2_/Si_3_N_4_ CMP: Removal rate selectivity, morphology, and mechanism research. J. Mol. Liq..

[2] Lei H., Luo J. (2004). CMP of hard disk substrate using a colloidal SiO_2_ slurry: Preliminary experimental investigation. Wear.

[3] Basim G.B., Vakarelski I.U., Moudgil B.M. (2003). Role of interaction forces in controlling the stability and polishing performance of CMP slurries. J. Colloid Interface Sci..

[4] Park S.-J., Lee H.-S., Jeong H. (2015). Signal analysis of CMP process based on AE monitoring system. Int. J. Precis. Eng. Manuf. Technol..

[5] Singh R.K., Lee S.-M., Choi K.-S., Basim G.B., Choi W., Chen Z., Moudgil B.M. (2002). Fundamentals of Slurry Design for CMP of metal and dielectric materials. MRS Bull..

[6] Hussain A., Jabeen N., Tabassum A., Ali J. (2024). 3D-Printed Conducting Polymers for Solid Oxide Fuel Cells, Chapter 12. 3D Printed Conducting Polymers.

[7] Ein-Eli Y., Starosvetsky D. (2007). Review on copper chemical–mechanical polishing (CMP) and post-CMP cleaning in ultra large system integrated (ULSI)—An electrochemical perspective. Electrochim. Acta.

[8] Moon Y. (2022). 1-Chemical and physical mechanisms of dielectric chemical mechanical polishing (CMP). Advances in Chemical Mechanical Planarization (CMP).

[9] Seo J., Lee J.W., Moon J., Sigmund W., Paik U. (2014). Role of the surface chemistry of ceria surfaces on silicate adsorption. Appl. Mater. Interfaces.

[10] Luan X., Liu Y., Zhang B., Wang S., Niu X., Wang C., Wang J. (2017). Investigation of the barrier slurry with better defect performance and facilitating post-CMP cleaning. Microelectron. Eng..

[11] Oliver M.R. (2004). Chemical-Mechanical Planarization of Semiconductor Materials.

[12] Kim J., Kwak D., Park J., Kubota T., Kim T. (2022). Effects of aging time in hydrogen peroxide-glycine-based Cu CMP slurry. Mater. Sci. Semicond. Process..

[13] Oh M.-H., Nho J.-S., Cho S.-B., Lee J.-S., Singh R.K. (2011). Polishing behaviors of ceria abrasives on silicon dioxide and silicon nitride CMP. Powder Technol..

[14] Basim G.B. (2011). Effect of slurry aging on stability and performance of chemical mechanical planarization process. Adv. Powder Technol..

[15] Seo Y.J., Kim S.Y., Choi Y.O., Oh Y.T., Lee W.S. (2004). Effects of slurry filter size on the chemical mechanical polishing (CMP) defect density. Mater. Lett..

[16] Basim G.B., Moudgil B.M. (2002). Effect of soft agglomerates on CMP slurry performance. J. Colloid Interface Sci..

[17] Kim J.-Y., Han S.-J., Kim S.-S. (2010). The enhanced electrophoresis method in leachate system for repairing of leaks in waste land fill geo membrane liner. J. Korean Soc. Civ. Eng..

[18] Song G.D., Kim M.H., Lee Y.T., Maeng W.Y. (2013). Improvement in the dispersion stability of iron oxide (Magnetite, Fe_3_O_4_) particles with polymer dispersant injection. Appl. Chem. Eng..

[19] Chiu W.-L., Huang C.-I. (2023). Polymer nanoparticles applied in the CMP (Chemical Mechanical Polishing) process of chip wafers for defect improvement and polishing rate response. Polymers.

[20] Zheng Y., Wang N., Feng Z., Tan X., Zhang Z., Han H., Huang X. (2022). The effects of precursors on the morphology and chemical mechanical polishing performance of ceria-based abrasives. Materials.

[21] Zhao L., Feng K., Zhao T., Zhou Z., Ding J. (2022). The preparation and performance analysis of a Cr_2_O_3_ gel abrasive tool for sapphire substrate polishing. Lubricants.

[22] Kim S.-I., Jeong G.-P., Lee S.-J., Lee J.-C., Lee J.-M., Park J.-H., Bae J.-Y., Park J.-G. (2021). Scavenger with protonated phosphite ions for incredible nanoscale ZrO_2_-abrasive dispersant stability enhancement and related tungsten-film surface chemical–mechanical planarization. Nanomaterials.

[23] Hwang S., Kim W. (2024). Characterization of ceria nanoparticles as abrasives applied with defoaming polymers for CMP (chemical mechanical polishing) applications. Polymers.

[24] Lee S.B., Park H.H., Bae I.S., Yoon J.S., Kim B.J. (2002). Effect of Al, Al_2_O_3_ dispersants and heat treatment on deposits from watt’s Ni plating bamth. Korean J. Mater. Res..

[25] Lyklema J., van Leeuwen H.P., Minor M. (1999). DLVO-theory, a dynamic re-interpretation. Adv. Colloid Interface Sci..

[26] Singh B.P., Menchavez R., Takai C., Fuji M., Takahashi M. (2005). Stability of dispersions of colloidal alumina particles in aqueous suspensions. J. Colloid Interface Sci..

[27] Choi S.W., Kim J. (2021). Facile room-temperature synthesis of cerium carbonate and cerium oxide nano- and microparticles using 1,1′ carbonyldiimidazole and imidazole in a nonaqueous solvent. J. ACS Omega.

[28] Hua L., Su Y., Wang X.-L., Helal M.-H., Ibrahim M., Huang M., El-Bahy S.-M., Jiang Q. (2022). Preparation and properties of ethylene–acrylic acid co polymer/Surlyn-Zn/zinc stearate blends ionic interlayer membrane. Adv. Compos. Hybrid Mater..

[29] Jadranka B.-G., Subrt J., Bastl Z., Pola J. (2006). IR laser ablative modification of poly(ethylene-co-acrylic acid) zinc salt. Polym. Degrad. Stab..

[30] Liu M., Zhang B., Seo J., Xian W., Cui D., Wu P., Wang Y., Liu S. (2004). Development of Highly Stable Ceria Slurry in Acetic Acid-Ammonium Acetate Buffer System for Effective Silica Chemical Mechanical Planarization. Mater. Sci. Semicond. Process..

[31] Han X., Liu R., Tan B., Wang F., Yan M., Zhao X., Zhao J. (2023). Research progress on the application of ceria nanoparticles as abrasives in dielectric layer CMP and post cleaning: Structure, morphology, doping, and mechanism. Colloids Surf. A Physicochem. Eng. Asp..

